# Synthesis and characterization of titanium complex with a dithiolate ligand for green LCD color filter dyes

**DOI:** 10.1186/1556-276X-7-635

**Published:** 2012-11-21

**Authors:** Hwangyu Shin, Youngil Park, Seungho Kim, Byeong-Kwan An, Jongwook Park

**Affiliations:** 1 Department of Chemistry, The Catholic University of Korea, Jibong-ro 43, Bucheon, 420-743, South Korea

**Keywords:** Bis(cyclopentadienyl) titanium complex, Pigment, Dyes, Molar extinction coefficiency, Color filter, Black matrix additive, Ligand-to-ligand charge transfer.

## Abstract

Three green compounds for color filter dyes based on bis(cyclopentadienyl) titanium complexes including dithiolate ligand were synthesized. Physical properties by the change of the substitution groups of the synthesized materials were systematically examined. UV-visible absorption spectrum of the synthesized materials showed maximum absorbing wavelengths of 427 to 430 nm and 632 to 635 nm in solution state, and 434 to 438 nm and 637 to 651 nm in film state, indicating green and black colors. It was observed that the extinction coefficient values (log *ε*) of all the synthesized materials are very high at 4.0 or above. In addition, it was shown that since the *T*_d_ values of three synthesized materials show thermal stability higher than 240°C, they possess high potential to be applied as dyes for LCD color filter and black matrix addictive.

## Background

Recently, thin-film transistor liquid crystal display (TFT LCD) panels have become increasingly important in the flat display panel industry [1]. Among major parts in TFT LCD panels, red, green, and blue (RGB) color filters are a very essential component for rendering full color images in TFT LCD panels. Various types of dyes and pigments for RGB color filters have been extensively developed and commercially utilized in manufacturing color filters of TFT LCD panels. So far, a wide range of blue and red color filter materials has been developed. However, a limited number of dyes and pigments for pure green color filters have been introduced because it is difficult to synthesize dye and pigment molecules which simultaneously absorb the light of both violet (<450 nm) and red (>600 nm) regions, but not that of around 500-nm region.

In order to manufacture a color filter for large-sized TFT LCD panels, the ink-jet printing technology has been extensively used since it affords a facile and economic production process. To apply ink-jet printing technology, dye-type color filter materials are preferred to pigment-type compounds because dye color filters are cheaper and have an easy control of fabrication process and a superior chromatic property, whereas the ink-jet nozzle is often blocked by insoluble pigment particles. However, dye-type color filter materials are needed to overcome the poor thermal stability for the high durability of TFT LCDs.

**Figure 1:**
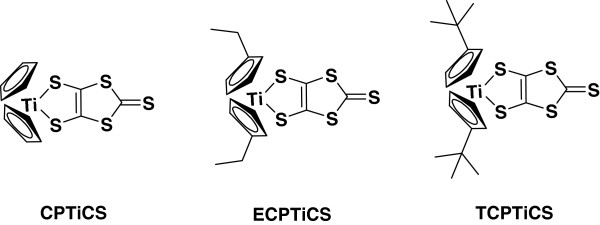
Chemical structures of bis(cyclopentadienyl)titanium complex derivatives.

In this work, we report a new class of green color filter materials of Cp_2_-Ti-dmit and its derivatives (see Figure 1), consisting of a titanium (IV) metal, a dithiolate ligand, and different cyclopentadienyl ligands. These titanium complexes are readily prepared by a two-step process and have good solubility in common organic solvents. The prepared titanium complex molecules show a green color with a high molar extinction coefficient (*ε*) at around 430 and 630 nm, and also exhibit a high thermal stability [1].

## Methods

### General method

^1^H-NMR spectra were recorded on Avance III 300 (Bruker BioSciences Korea Co. Ltd., Seoul, South Korea). Fast atom bombardment (FAB) mass spectra were recorded by JMS-AX505WA, HP5890 series II (Jeol Korea Ltd., Seoul, South Korea). The optical absorption spectra were obtained by HP 8453 UV-vis-NIR spectrometer (Hewlett-Packard Asia Pacific Ltd., Singapore). Degradation temperatures (*T*_d_) of the compounds were measured by thermogravimetric analysis (TGA) using a SDP-TGA2960 (TA Instruments, DE, USA).

### Synthesis of tetraethylammonium bis(1,3-dithiole-2-thione-4,5-dithiol)zincate (ZnCS_2_)

Three-liter round flask was charged with sodium shaving (2.3 g, 0.1 mol) via a solid addition funnel and, after reassembly, the apparatus was flushed with N_2_ for 5 min and placed in ice-water bath. Carbon disulfide (18 ml, 0.3 mol) was introduced into the flask through the dropping funnel. After which, the dropping funnel was immediately loaded with 20 ml dimethylformamide (DMF), the system was closed, and N_2_ atmosphere was maintained throughout the reaction. The DMF was added dropwise with stirring over 1 hr, during which, a red color appears. Upon completion of the addition, the temperature of the reaction mixture was allowed to warm to room temperature. The mixture was stirred overnight, during which time it becomes increasingly deep red. The reaction mixture was visually inspected for residual sodium. As a precaution, ice was added to the cooling bath and 40 ml methanol and 50 ml deionized water, which is degassed by applying vacuum, were then added rapidly through the dropping funnel. A solution of zinc chloride (2 g, 0.15 mol) in a mixture of 40-ml concentrated ammonium hydroxide and 50 ml of methanol was then added through the dropping funnel. A solution of tetraethylammonium bromide (5.3 g 0.025 mol) in 25 ml of deionized water was added dropwise via the dropping funnel with vigorous stirring over at least 1 h, and the solution was stirred overnight [2]. A large amount of red precipitate develops in the flask overnight. This salt was collected by solution on a Buchner funnel and was washed immediately with 50 ml of deionized water, then with 40 ml portion of isopropyl alcohol until the filtrate is colorless, and finally once with 20 ml of diethyl ether. After completion of the reaction, the solvent was evaporated under vacuum, and the product was extracted with dichloromethane (CH_2_Cl_2_). The CH_2_Cl_2_ solution was washed with water and dried with magnesium sulfate anhydrous. The solvent was evaporated to give ZnCS_2_ as red solid, which was recrystallized from CH_2_Cl_2_ and hexane (yield 81%).

### Synthesis of Bis(cyclopentadienyl)titanium complex (CPTiCS)

Treatment of Bis(cyclopentadienyl)Titanium(IV) dichloride (1g, 4mmol) with 0.6 equiv of ZnCS_2_ in refluxing tetrahydrofuran(THF) resulted in a color change red to green. After 4 h, the cooled solution was filtered through a silica gel, concentrated, and diluted with hexane to afford deep green crystals of CP_2_TiCS [3] (yield 40%). ^1^H-NMR (300 MHz, DMSO) 6.12 (s, 10H). C_13_H_10_S_5_Ti, found C, 42.73; H, 2.62; S, 42.87; calculated C, 41.70; H, 2.69; S, 42.82; FAB-mass: 374 m/z.

### Synthesis of bis(ethylcyclopentadienyl)titanium complex (ECPTiCS)

Treatment of bis(ethylcyclopentadienyl)titanium(IV) dichloride (0.5 g, 1.6mmol) with 0.6 equivalents of ZnCS_2_ in refluxing THF resulted in a color change from red to green. After 6 h, the cooled solution was filtered through a silica gel, concentrated, and diluted with hexane to afford black crystals of ECP_2_TiCS (yield 41.8%). ^1^H-NMR (300 MHz, DMSO), 6.15 (d, 8H), 2.36 (m, 4H), 1.01 (t, 6H). C_17_H_18_S_5_Ti, found C, 47.52; H, 4.24; S, 37.17; calculated C, 47.43; H, 4.21; S, 37.24; FAB-mass 430 m/z.

### Synthesis of bis(tetrabutlycyclopentadienyl)titanium complex (TCPTiCS)

Treatment of bis(tetrabutlycyclopentadienyl)titanium(IV) dichloride (0.9 g, 2.5 mmol) with 0.6 equivalents of ZnCS_2_ in refluxing THF resulted in a color change from red to green. After 4 h, the cooled solution was filtered through silica gel, concentrated, and diluted with hexane to afford deep green crystals of TCP_2_TiCS (yield 40%). ^1^H-NMR (300 MHz, DMSO) 6.13 (d, 8H), 1.12 (s, 18H). C_21_H_26_S_5_Ti, found C, 51.89; H, 5.49; S, 32.98; calculated C, 51.83; H, 5.39; S, 32.95; FAB-mass 486 m/z.

### Solubility of ECPTiCS, bis(cyclopentadienyl)titanium complex (CPTiCS), and TCPTiCS

For solubility test, 0.1 g of compound and 0.4 g of PGMEA were mixed and stirred for 20 h. After stirring, syringe filter (membrane material: Teflon) was used for filtering. Then, the weight of the specimen on the membrane was measured by the difference before/after filtering [4].

## Results and discussion

**Figure 2:**
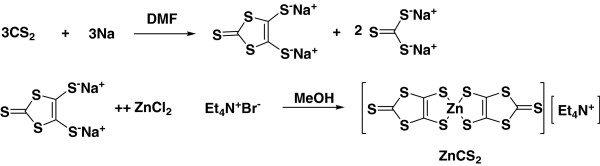
Synthetic route of ZnCS_2_.

**Figure 3:**
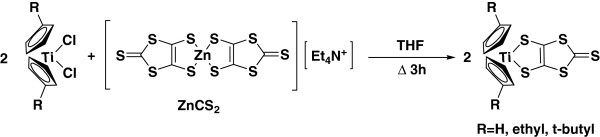
Synthetic route of CPTiCS (R = H), ECPTiCS (R = ethyl), and TCPTiCS (R = t-butyl).

The structure of new dye based on dithiolate ligands and cyclopentadienyl ligand is shown in Figure 1. Sodium, disulfate, and DMF were reacted in deionized water to synthesize ZnCS_2_ as the intermediate stage for black-green dye synthesis, and the synthesis method is shown in Figure 2. The synthesized ZnCS_2_ was dissolved in THF solvent to synthesize with bis(cyclopentadienyl)titanium (IV) dichloride derivatives as shown in Figure 3.

**Figure 4:**
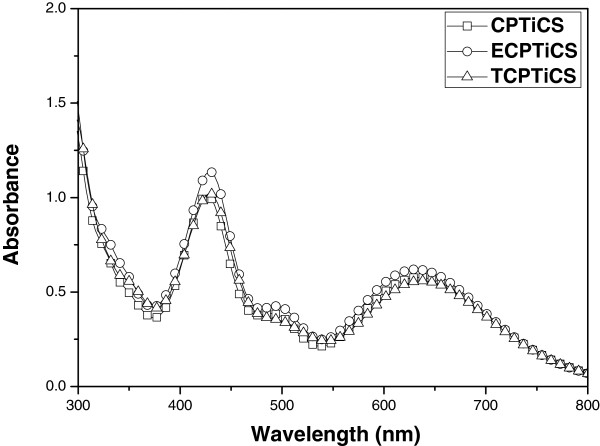
**UV-visible absorption spectra.** CPTiCS (square), ECPTiCS (circle), and TCPTiCS (triangle) in THF solution (1 × 10^−4^ M).

**Figure 5:**
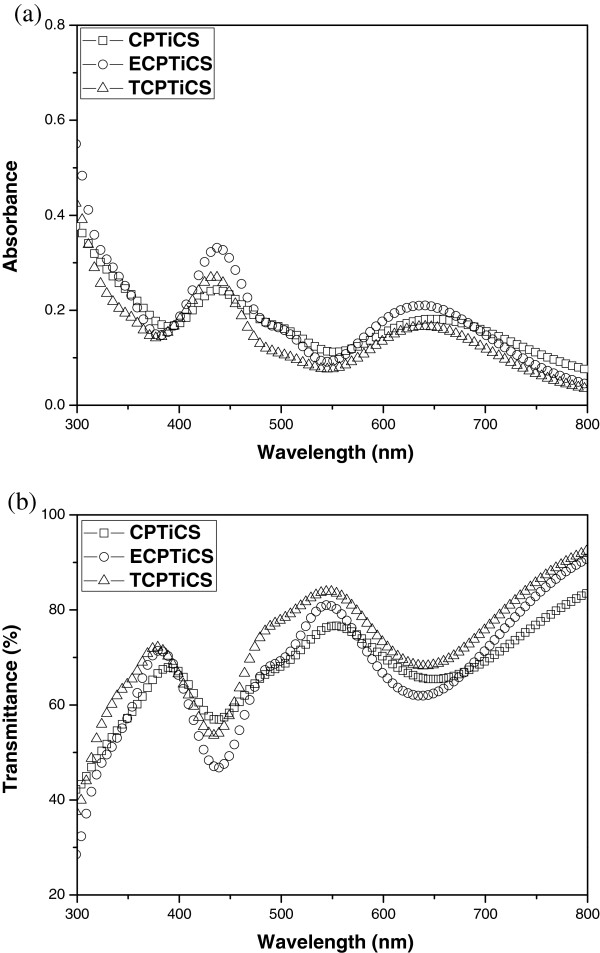
**UV-visible absorption (a) and transmittance (b) spectra.** CPTiCS (square), ECPTiCS (circle), and TCPTiCS (triangle) in film state. Film thickness is 40 nm.

The optical property of the synthesized compound was checked using UV-visible (UV-vis) absorption spectrum, and it is described in Figures 4 and 5 and in Table 1. First, UV-vis absorption spectra of CPTiCS and ethyl group-substituted ECPTiCS, and t-butyl group-substituted TCPTiCS were measured in THF solution state (1 × 10^−4^ M). For all three compounds, the maximum absorption wavelength appeared near 430 nm in violet area and another peak near 630 nm of the red region. In case of ECPTiCS and TCPTiCS where ethyl group and t-butyl group are substituted, the intensity of maximum peak and the second maximum peak was found to be very slightly increased compared to CPTiCS. Also, from UV-vis absorption spectra of the films made by spin-coating method, the maximum absorption wavelength of CPTiCS appeared to be about 436 nm (ECPTiCS) and 651 nm (TCPTiCS). In case of ECPTiCS and TCPTiCS, the maximum absorption wavelength appeared 438 and 434 nm, respectively, and another peak of 637 and 638 nm with about 5 nm of red shift from solution state. In case of CPTiCS, there is no bulky substituent to increase the intermolecular interaction in bis(cyclophentadiene) ligand, so the film-state UV-vis absorption spectra appeared to be vivid red-shifted for 16 nm compared to ECPTiCS and TCPTiCS (see Table 1). The synthesized dyes appeared green in THF solution and deep green in powder.

**Table 1:** Optical properties of synthesized materials

**Compound**	**SolutionUV**_**max**_**(nm)**	**FilmUV**_**max**_**(nm)**	log *ε *(L/mol*cm)	**Solution color**
CPTiCS	427,635	436,651	4.00	Green
ECPTiCS	428,632	438,637	4.07	Green
TCPTiCS	429,633	434,638	4.01	Green

In ligand-metal-ligand complex synthesized in this study, dithiolate ligand was used as π-electron donor, and bis(cyclopentadienyl) group was used as π-electron acceptor. Thiolate unit which is electron rich and bis(cyclopentadienyl) unit which is relatively electron lacking were attached on titanium(IV) center metal in one molecule. This induces intramolecular ligand-to-ligand charge transfer (LLCT) [5,6]. As a result, the light of blue and red area is absorbed, and it eventually shows green-colored UV-absorption spectra [7] (Figure 5).

The absorption band in the range of 400 to 500 nm (blue absorption region) and 600 to 800 nm (red absorption region) is attributed to the π-π* transition of 1,3-dithiole-2-thione-4,5-dithiolato (dmit) ligand and ‘intramolecular’ LLCT, respectively [7,8]. Regarding the LLCT process, the charge transfer (CT) band is presumably originated from electron-rich dmit ligand to the cyclopentadieneyl electron acceptor which is also often observed in the metal complex system containing both a dmit ligand as an electron donor and a bypyridine ligand as a π-electron acceptor [5-7].

The three synthesized dyes were measured for *ε* under the conditions of THF solvent. As the result, all synthesized substances showed high log scale *ε* value of over 4.0. The properties of materials with high *ε* values and absorbing 300 to 800 nm at the same time confirmed that it can be used not only as green dye for LCD color filter but also as the additive substance used for black matrix. As mentioned the above, the absorption bands of Ti complex dyes mainly originated from a mixture of π-π* transition and LLCT, owing to the dmit ligand. In general, it is well known that π-π* and CT transitions have high molar extinction coefficients (>10,000 L/mol·cm), leading to the intense absorption bands, whereas n-π* and d-d transitions exhibit a low molar extinction coefficients, *ε* = 10 to 100 and *ε* = 5 to 500 L/mol·cm, respectively [5-8].

**Figure 6:**
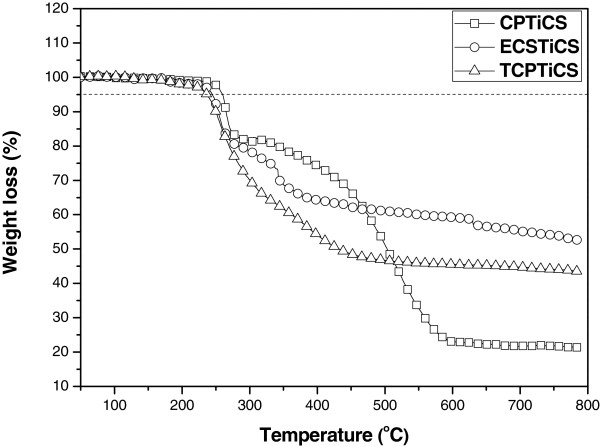
TGA thermal properties of CPTiCS (square), ECPTiCS (triangle), and TCPTiCS (circle).

**Figure 7:**
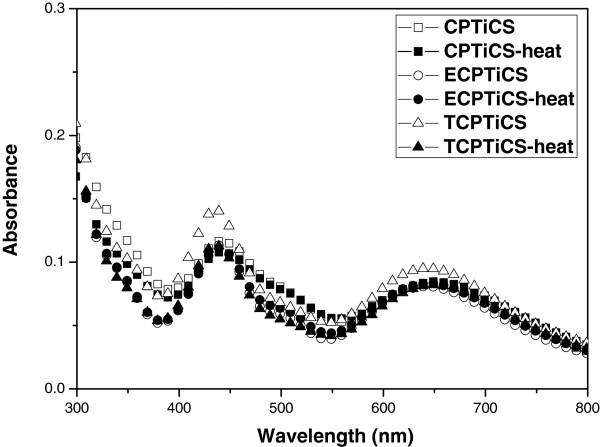
**Thermal stability of spin-coated dye films (bold style).** The changes of absorption spectra of spin-coated Ti complex films before and after heat treatment at 200°C for 30 min.

For the dyes used in LCD, not only absorption wavelengths and *ε*[1] but also thermal stability and solubility are important [4]. Although the dyes have superior optical properties than the pigments, they were hardly applied for LCD color filters owing to their inferior thermal stability. For dyes to be used as color filters, they should endure a temperature of 220°C, which is the current highest temperature in the LCD manufacturing process, without significant weight loss [9,10]. As the result of TGA measurement, all three substances showed *T*_d_ value of over 240°C, confirming the thermal stability (see Figure 6). For TGA measurement, the TGA curves were obtained with a heating rate of 10°C/min under nitrogen. In terms of the thermal stability of the spin-coated dye films on color changes, it was found that there is no significant change of absorption spectra before and after heating treatment of the spin-coated dye films (prepared by 0.5wt% solution) at 200°C for 30 min (see Figure 7). The synthesized Ti complexes showed high solubility in most organic solvents such as THF, chloroform, and toluene. Moreover, 1-methoxy-2-propanal acetate (PGMEA), the solvent commercialized in color filer, was used to execute solubility test, and as the result, CPTiCS showed about 10% of solubility, but ECPTiCS and TCPTiCS showed high solubility of over 20% due to the substituent effect of alkyl group introduced for solubility increase (see Table 2).

**Table 2:** Optical and thermal properties of synthesized materials

**Compound**	**Solubility (PGMEA) (%)**	***T***_**d**_**(°C)**^**a**^
CPTiCS	11	265
ECPTiCS	26	250
TCPTiCS	27	241

^a^*T*_d_: decomposition temperature (5% weight loss).

## Conclusions

A new class of green dyes based on bis(cyclopentadienyl) titanium complexes, CPTiCS, ECPTiCS, and TCPTiCS, were designed and synthesized for LCD color filters. All prepared green dyes were soluble in a common color filter solvent, PGMEA, (over 10 wt% of solubility) and exhibited distinctive absorption bands around at 430 nm and 630 nm with a high molar extinction coefficient (log *ε* > 4.0) owing to the π-π* transition and ligand-to-ligand charge transfer (LLCT), respectively. In addition they showed a high thermal stability with 5% weight-loss temperature beyond 241°C and their spin-coated films had no significant change of absorption spectra after heating treatment at 200°C for 30 min.

## Competing interests

The authors declare that they have no competing interests.

## Authors’ contributions

HS carried out the main synthesis of the synthesized compounds. YP carried out the minor synthesis of the synthesized compounds. SK carried out the thermal treatment experiment of the spin-coated films. BKA raised the idea of final chemical structures. JP suggested the synthetic routes and characterization methods of the synthesized compounds. All authors read and approved the final manuscript.

## Authors’ information

HS is a masters degree student on Organic Material Chemistry. YP is a Ph.D. degree holder on Organic Material Chemistry and is currently a postdoctoral fellow. SK is a masters degree student on Organic Material Chemistry. BKA is a Chemistry professor and is majoring in Analytical Chemistry. JP is a full professor on Organic Material Chemistry. He is also a director of Display Research Center of the Catholic University in South Korea.
